# Neck dissection complications

**DOI:** 10.1590/S1808-86942011000100011

**Published:** 2015-10-19

**Authors:** Rogério Aparecido Dedivitis, André Vicente Guimarães, Elio Gilberto Pfuetzenreiter, Mario Augusto Ferrari de Castro

**Affiliations:** 1 Associate professor, Lusíada Foundation - UNILUS, Physician; 2 Doctoral degree, Surgery Department, Medical School of the São Paulo University, São Paulo/SP. Assisting at the Head & Neck Surgery Unit of the Ana Costa Hospital and the Holy House of Mercy Sisterhood in Santos, Santos/SP, Brazil; 3 Master's degree in Sciences, graduate course of the Heliópolis Hospital, São Paulo/SP. Professor in the Surgery Department, Lusíada Foundation - UNILUS, Santos. Assisting at the Head & Neck Surgery Unit of the Ana Costa Hospital and the Holy House of Mercy Sisterhood in Santos, Santos/SP, Brazil; 4 Medical resident of head & neck surgery, Ana Costa Hospital, Santos. Professor in the Surgery Department, Fundação Lusíada UNILUS, Santos/SP, Brazil

**Keywords:** intraoperative complications, postoperative complications, neck dissection

## Abstract

Because of the proximity of vital structures, certain complications are inherent to neck dissection (ND) for the treatment of patients with squamous cell carcinoma of the upper aerodigestive tract.

**Aim:** To establish the incidence of complications of ND.

**Methods:** A cross-sectional retrospective study of patient registries. ND with curative intention was evaluated in 480 patients with squamous cell carcinoma of the upper aerodigestive tract from January 1995 to December 2008 to identify perioperative complications.

**Results:** Considering the total quantity of dissected neck sides, 413 radical ND and 295 selective ND were studied, of which 220 were supraomohyoid ND and 75 were jugular ND, totaling 708 sides. There were no deaths. The most frequent complication was marginal mandibular nerve injury (5.5%), followed by accessory nerve injury (5.1%). However, in 18 out of 21 cases this nerve was sacrificed for oncological completeness.

**Conclusions:** There were no perioperative deaths. Nerves were the most commonly injured structures; the marginal mandibular branch is injured most (5.5%).

## INTRODUCTION

Neck dissection (ND) is a procedure for diagnosing (staging) and treating head and neck cancer. It consists of removing lymph nodes from specific areas of the neck, and removing or not the sternocleidomastoid muscle, the internal jugular vein, and the accessory nerve. Neck lymph node excision not only helps stage the disease but also treats lymphatic disease and defines the need for adjuvant therapy, such as radiotherapy. Because vital anatomical structures are close, certain risks and complications are inherent to this procedure.^1^ It is important in these patients to preventing, recognize, and treat the early and late manifestations of ND.

Since Crile^2^ introduced radical ND at the beginning of the 20^th^ century, a few changes have been proposed, in particular Suárez's^3^ functional ND, which aimed for a more conservative approach to preserve vital anatomical structures in the neck without compromising the completeness of lymph node removal. The transition from radical to selective ND has resulted in fewer complications and lower morbidity, at the same time preserving surgical efficacy and compliance with oncologic principles.^4^

Chemotherapy has been investigated as an alternative approach to primary surgical resection with the aim of preserving organs in patients with advanced head and neck tumors. Neck metastatic disease is one of the most significant prognostic factors; metastases generally respond less to organ preservation protocols than primary tumors. Thus, planned and salvage ND after chemotherapy^5^ has been proposed for patients with advanced regional disease, although there is a possible higher rate of complications.

The purpose of this study was to establish the incidence of complications in patients with squamous cell carcinoma of the upper aerodigestive tract after ND therapy.

## METHOD

ND with a curative intent was done in 480 patients with squamous cell carcinoma of the upper aerodigestive tract (434 male and 46 female) from January 1995 to December 2008. A cross-sectional retrospective study was made of the medical files of these patients. The mean age of patients was 56 years, ranging from 33 to 75 years. Table 1 shows the distribution by primary sites. The first treatment at our hospital is an association of radiotherapy and chemotherapy done simultaneously when the primary site is the nasopharynx (lymphoepithelioma). All cases with disease at this site underwent salvage ND for neck recurrences; these were stratified with cases treated with organ preservation protocols. Table 2 shows the type and number of NDs.

**Table 1:** Distribution of patients according to the primary site of the disease

Time of neck dissection	Primary site	Number of patients
Primary treatment	Mouth	219
	Oropharynx	52
	Larynx	97
	Hypopharynx	19
	Paranasal sinuses	6
	Skin	2
	Hidden primary	41
	Total	436
	Oropharynx	23
After organ preservation protocol or salvage of nasopharyngeal recurrence	Larynx	10
	Hypopharynx	5
	Nasopharynx	6
	Total	44

**Table 2:** Types of neck dissections (ND)

Type of ND	Number of patients
Unilateral selective	113
Bilateral selective	42
Unilateral radical	139
Contralateral radical + selective	98
Bilateral radical	88

Indications for ND depended on neck staging (N): selective ND was done when evident disease was absent or only minimal neck disease was present (N1 necks in selected cases of mouth cancer); classical and modified radical ND was done if there was clinically evident neck adenopathy, preserving non-lymphatic neck structures (accessory nerve and internal jugular vein e internal jugular vein) as long as surgical completeness was not compromised. Selective or radical bilateral ND was indicated if contralateral disease was suspected or present, based on the abovementioned criteria for each side. Adjuvant radiotherapy was indicated if there were histopathologically confirmed metastases or depending on primary tumor extension or aggressiveness criteria. Concomitant adjuvant radiotherapy and chemotherapy was indicated if extracapsular invasion was documented, and in all N3 cases.

The following complications were investigated: suture line dehiscence (epidermolysis; and deep dehiscence); infection/suppuration; chylous leakage; intraoperative and postoperative hemorrhage; subcutaneous emphysema; pneumothorax; salivary leakage; cephalic vein stasis (manifested as edema/facial cyanosis, suffusion/conjunctival edema; proptosis; venous engorgement of retina; and seizures/cardio-respiratory depression); bronchopneumonia and lung complications; nerve injury (accessory nerves, marginal mandibular branch, vagus, hypoglossal, phrenic nerves, and the cervical sympathetic trunk); uncommon complications (arteriovenous fistula, inappropriate ADH secretion syndrome, cerebral vascular accident, air embolism, and blindness); and intraoperative mortality.

## RESULTS

The total number of sides that were studied was 708, of which 413 were radical NDs and 295 were selective NDs; of the latter, 220 were supraomohyoid NDs and 75 were jugular NDs. Table 3 presents the incidence of complications. There were no intraoperative deaths. The most frequent complication was injury of the marginal mandibular branch, which was encountered in 39 sides undergoing ND (5.5%). This was followed by injury of the accessory nerve, which was observed in 21 of 413 sides in which radical ND was done (5.1%); of these, however, the nerve was sacrificed in 18 cases for oncologic completeness.

**Table 3:** Complication of neck dissection (ND).

Complication	Unilateral selective ND (n = 113)	Bilateral selective ND (n = 42)	Unilateral radical ND (n = 139)	Radical + selective ND (n = 98)	Bilateral radical ND (n = 88)
Intraoperative hemorrhage	0	0	0	0	0
Postoperative hemorrhage	0	0	1	0	0
Epidermolysis	0	0	15	13	15
Deep dehiscence	0	0	3	4	4
Wound infection	0	0	1	0	1
Chylous leakage	0	0	0	1	2
Subcutaneous emphysema	0	0	0	0	0
Pneumothorax	0	0	0	0	0
Salivary leakage	0	0	0	0	0
Cephalic vein stasis	0	0	0	0	6
Bronchopneumonia	0	1	1	2	2
Accessory nerve injury	0	0	9	7	5
Marginal mandibular nerve injury	6	5	8	11	9
Vagus nerve injury	0	0	0	0	0
Hypoglossal nerve injury	0	0	0	1	2
Phrenic nerve injury	0	0	0	0	1
Sympathetic trunk injury	0	0	1	0	2
Uncommon complications	0	0	0	0	1*
Intraoperative mortality	0	0	0	0	0

(*) bilateral blindness

## DISCUSSION

Although ND is a technically well-established procedure, complications still occur. Intraoperative events, such as hemorrhage, loss of a venous suture resulting in gas embolism, chylous leakage due to thoracic duct injury, and arrhythmia because of carotid bulb manipulation, are habitually promptly managed; these events may, however, be disastrous for the patient. Careful dissection and ligature of vessels are extremely important to avoid intra-and postoperative hemorrhage. Hematomas are avoided by careful hemostasis and continuous suction drainage.^6^

Prior radiotherapy affects post-ND healing. This effect is dose-dependent; higher doses result in more extensive fibrosis, hypoxia, and decreased leukocyte migration.^7^ There were 43 cases of epidermolysis, mostly in the “T” intersection of the flaps, which were treated conservatively. There were also 11 cases of deep dehiscence, five of which were managed by rotating the pedicled flap. There were no significant differences between previously irradiated cases and those undergoing surgery primarily.

The surgical wound infection rate was low; preventive antibiotic therapy was given in the preceding 24 hours to surgery. Extensive vascularization of the neck and the uncontaminated nature of the neck surgical field explain this low rate of infection. Factors favoring infection are contamination of the neck when laryngectomy or tracheotomy are done; previous radiotherapy, however, had no negative impact.^8^ There were two cases of surgical wound infection, one in a patient that had undergone radiotherapy before surgery. A protective factor against neck contamination and surgical wound infection is the use of staplers for constructing the neopharynx after total laryngectomy.^9^

Chylous leakage is rare; it occurs in 1 to 2.5% of NDs, mostly in the left side. The more easily accessible portion of the thoracic duct is located along the medial aspect of the internal jugular vein; consequently, this is the most common injury site. Positive ventilation pressure maneuvers may help locate and repair this injury if it is found during the procedure.^10^ There were three cases following radical ND; one was treated conservatively (nothing per orum and compressive dressings), and two were reoperated.

An increased complication rate is associated with planned or salvage ND after the organ preservation protocol. These include dehiscence and flap necrosis, especially in pharyngeal and laryngeal procedures, where the possibility of associated pharyngocutaneous leakage is higher; this may result in an increased hospital stay and additional surgeries.^6,^^11^ There was one case of postoperative hemorrhage; this patient was reoperated, the hematoma was drained, the flaps were irrigated and a tributary of the internal jugular vein was ligated - its ligature had been lost.

Facial edema generally occurs when venous return is compromised after bilateral radical ND; rarely, it is massive and accompanied by cerebral edema and even death. We routinely attempt to preserve at least one of the internal jugular veins; nevertheless, this complication arose in six patients after bilateral radical ND. There were no cerebral complications.

Nerve injury during ND is not uncommon; it may result in loss of function or pain syndromes. The incidence is low after functional ND (in a 442 patient series): accessory nerve injury - 1.68%; marginal mandibular nerve injury - 1.26%; hypoglossal nerve injury - 0.56%; and sympathetic cervical nerve injury - 0.42%.^12^

Marginal mandibular never injury usually occurs when the upper flap is elevated or during level 1 dissection (submental/submandibular triangles); it may cause dysfunction of the lower lip depressor muscle. This injury was encountered in 18% of patients and 23% of necks in a study of 66 patients undergoing ND, resulting in an asymmetric smile but no severe sequelae.^13^

The vagus nerve may be injured when the internal jugular vein is ligated during radical ND. If below the nodose ganglion, it results in vocal fold paralysis; if above, there is also dysphagia and aspiration.^14^ This complication is rare, and was not encountered in our series. Phrenic nerve injury is uncommon; it often goes unnoticed, and may result in atelectasis and lung infiltrates.^15^ We found no cases of phrenic nerve injury.

Even when the accessory nerve is preserved, it is skeletonized (and therefore devascularized), which may result in the painful shoulder syndrome; it is not uncommon and affects the patient's quality of life. The surgeons should be familiar with the anatomy of the posterior triangle of the neck; nerve anastomosis or grafting may be beneficial if the nerve is transected or removed for oncologic reasons. A specific questionnaire of 65 patients in a study with a follow-up period over 1.6 years showed that 23% denied any shoulder dysfunction, 54% reported mild shoulder dysfunction, 15% reported moderate shoulder dysfunction, and 8% reported severe shoulder dysfunction.^16^

The Bernard-Horner syndrome or oculosympathetic paresis (blepharoptosis, enophthalmos, and miosis) is caused by injury of the cervical sympathetic nerve. This nerve is located posterior to and under the carotid sheath, and may be injured during radical ND radical;^17^ this occurred in three sides in our series.

Blindness is one of the most catastrophic of rarely described complications in the literature; 13 cases have been reported.^18^ It may be caused by cortical infarction, posterior ischemic optic neuropathy, or bilateral carotid occlusion; anemia, low arterial blood pressure and preexisting vascular disease are risk factors.^6^ There was one case of blindness after bilateral radical ND of no evident cause.

The indications of bilateral radical ND remain controversial. A study of 193 cases showed that the morbidity was high in these cases; it is therefore not indicated electively.^19^ There was a higher rate of complications in this group of patients in our series; there were, however, no cases of intraoperative mortality.

With the popularization of radiochemotherapy in the 1990s to preserve organs, and considering that neck metastasis is an independent prognostic factor, ND has been indicated as salvage or planned surgery after a radiochemotherapy protocol. However, surgical wound complication rates are about 10% when ND is undertaken from 5 to 17 weeks after the protocol; closure with pedicled flaps is required in 6% of cases.^20^ ND before radiochemotherapy has been suggested; the complication rate is 2.5%.^21^

## CONCLUSION

There were no intraoperative deaths. The most common lesions are nerve injuries; the mandibular marginal branch is the most commonly involved nerve (5,5%).

## References

[1] Smullen JL, Lejeune FE (1999). Complications of neck dissection. J La State Med Soc..

[2] Crile G (1906). Excision of cancer of the head and neck. With special reference to the plan of dissection based on 132 patients. JAMA..

[3] Suarez O (1963). El problema de las metastasis linfáticas y alejadas del cáncer de laringe e hipofaringe. Rev Otorrinolaringol..

[4] Ferlito A, Gavilán J, Buckley JG, Shaha AR, Miodonski AJ, Rinaldo A (2001). Functional neck dissection: fact and fiction. Head Neck..

[5] Wang SJ, Wang MB, Yip H, Calcaterra TC (2000). Combined radiotherapy with planned neck dissection for small head and neck cancers with advanced cervical metastases. Laryngoscope..

[6] Genden EM, Ferlito A, Shaha AR, Talmi YP, Robbins KT, Rhys-Evans PH (2003). Complications of neck dissection. Acta Otolaryngol..

[7] Mathes SJ, Alexander J (1996). Radiation injury. Surg Oncol Clin N Am..

[8] Coskun H, Erisen L, Basut O (2000). Factors affecting wound infection rates in head and neck surgery. Otolaryngol Head Neck Surg..

[9] Dedivitis RA, Guimarães AV (2004). Use of stapler for pharyngeal closure after total laryngectomy. Acta Cir Bras..

[10] de Gier HH, Balm AJ, Bruning PF, Gregor RT, Hilgers FJ (1996). Systematic approach to the treatment of chylous leakage after neck dissection. Head Neck..

[11] Dedivitis RA, Ribeiro KC, Castro MA, Nascimento PC (2007). Pharyngocutaneous fistula following total laryngectomy. Acta Otorhinolaryngol Ital..

[12] Prim MP, De Diego JI, Verdaguer JM, Sastre N, Rabanal I (2006). Neurological complications following functional neck dissection. Eur Arch Otorhinolaryngol..

[13] Batstone MD, Scott B, Lowe D, Rogers SN (2009). Marginal mandibular nerve injury during neck dissection and its impact on patient perception of appearance. Head Neck..

[14] Cece JA, Lawson W, Biller HF, Eden AR, Parisier SC (1987). Complications in the management of large glomus jugulare tumors. Laryngoscope..

[15] de Jong AA, Manni JJ (1991). Phrenic nerve paralysis following neck dissection. Eur Arch Otorhinolaryngol..

[16] Carr SD, Bowyer D, Cox G (2009). Upper limb dysfunction following selective neck dissection: a retrospective questionnaire study. Head Neck..

[17] Bucci T, Califano L (2008). Bernard-Horners syndrome: unusual complication after neck dissection. J Oral Maxillofac Surg..

[18] Worrell L, Rowe M, Petti G (2002). Amaurosis: a complication of bilateral radical neck dissection. Am J Otolaryngol..

[19] Magrin J, Kowalski L (2000). Bilateral radical neck dissection: results in 193 cases. J Surg Oncol..

[20] Ha PK, Couch ME, Tufano RP, Koch WM, Califano JA (2005). Short hospital stay after neck dissection. Otolaryngol Head Neck Surg..

[21] Prades JM, Timoshenko AP, Schmitt TH, Delolme MP, Francoz M, Martin C (2008). Planned neck dissection before combined chemoradiation for pyriform sinus carcinoma. Acta Otolaryngol..

